# Rare and heterogeneous manifestations of leucocyte adhesion deficiency type 1: report of two cases with diagnostic dilemmas and novel *ITGB2* mutation

**DOI:** 10.1186/s13223-023-00786-3

**Published:** 2023-05-02

**Authors:** Sabiha Anis, Aiysha Abid, Sadaf Aba Umer Kodwavwala, Sabahat Sarfaraz, Samina Junejo, Saba Shahid, Sajid Sultan, Adibul Hasan Rizvi

**Affiliations:** 1 Department of Pathology and Medicine & Allied, Section: Immunology, The Indus Hospital and Health Network (IHHN), Karachi, Pakistan; 2 Department of Human Genetics and Molecular Medicine, Sindh Institute of Urology and Transplantation (SIUT), Karachi, Pakistan; 3 Department of Pediatric Urology, Sindh Institute of Urology and Transplantation (SIUT), Karachi, Pakistan; 4 Department of Immunology, Department of Pathology, Dow International Medical College, Dow University of Health Science, Karachi, Pakistan; 5 Department of Pediatrics, The Indus Hospital and Health Network (IHHN), Karachi, Pakistan; 6 Department of Urology, Sindh Institute of Urology and Transplantation (SIUT), Karachi, Pakistan

**Keywords:** Primary immunodeficiency. Leucocyte adhesion defect, Novel ITGB2 variants, High TLC counts

## Abstract

**Background:**

Primary immunodeficiency disorders (PID) are rare disorders with heterogeneous manifestations, overlapping with other diseases such as autoimmunity, malignancy, and infections. This makes the diagnosis very challenging and delays management. Leucocyte adhesion defects (LAD) are a group of PIDs in which patients lack adhesion molecules on leukocytes needed for their emigration through blood vessels to the site of infection. Patients with LAD can present with diverse clinical features including severe and life-threatening infections, early in life, and the absence of pus formation around infection or inflammation. There is often delayed umbilical cord separation, omphalitis, late wound healing, and a high white blood cell count. If not recognized and managed early, can lead to life-threatening complications and death.

**Case Presentation:**

LAD 1 is characterized by homozygous pathogenic variants in the integrin subunit beta 2 (*ITGB2)* gene. We report two cases of LAD1 with unusual presentations (post-circumcision excessive bleeding and chronic inflammation of the right eye) which were confirmed by flow cytometric analysis and genetic testing. We found two disease-causing *ITGB2* pathogenic variants in both cases.

**Conclusions:**

These cases highlight the importance of a multidisciplinary approach to recognizing clues in patients with uncommon manifestations of a rare disease. This approach initiates a proper diagnostic workup of primary immunodeficiency disorder leading to a better understanding of the disease, and appropriate patient counseling, and helps clinicians to be better equipped to deal with complications.

## Background

Primary immunodeficiency disorders (PID) are rare diseases with an overall global prevalence of 0.02–0.1%. There are more than 400 PIDs that have been identified so far. The clinical features of these disorders are mostly non-specific and overlap with features of infections, autoimmunity, inflammation, allergies, malignancies, etc. [1]. Because of the overlapping symptoms and lack of awareness about these rare disorders, there is a tendency to miss these diseases and thus hampers or delays proper patient management [2].

Leucocyte adhesion defects (LAD) are a group of PIDs, characterized by the absence of adhesion molecules on leucocytes resulting in an inability to migrate to the site of infection and inflammation. This results in recurrent severe infections, mostly early in life, leading to failure to thrive and death if not recognized and managed early [3, 4]. LADs are classified into types-I, II, and III, categorized by the presence of homozygous pathogenic variants in the *ITGB2*, *SLC35C1*, and *FERMT3* genes, respectively [3, 5–8]. Recently a type 4 LAD has been described as characterized by pathogenic variants in the transmembrane conductance *regulator* (*CFTR*) gene. In LAD 4, the defects are present mainly in monocytes, unlike in LAD I-III, where the main defects are in the neutrophils [3, 9]. LAD can be presented with diverse clinical features. However, the common features in all cases are characterized by a high total leucocyte count (TLC) count in the blood due to their inability to move out to the site of infection and the absence of pus formation around infection or inflammation [6, 10].

Here we report two cases of leukocyte adhesion deficiency type1 with heterogeneous manifestations. The first case was a four-month-old boy, who presented to us with post-circumcision bleeding and did not respond to surgical measures. The second case was of an eight months old female child who presented with a granulomatous lesion of the right eye with a novel homozygous disease-causing variant identified in the *ITGB2* gene.

### Case presentations

#### Four-month-old

**Fig. 1:**
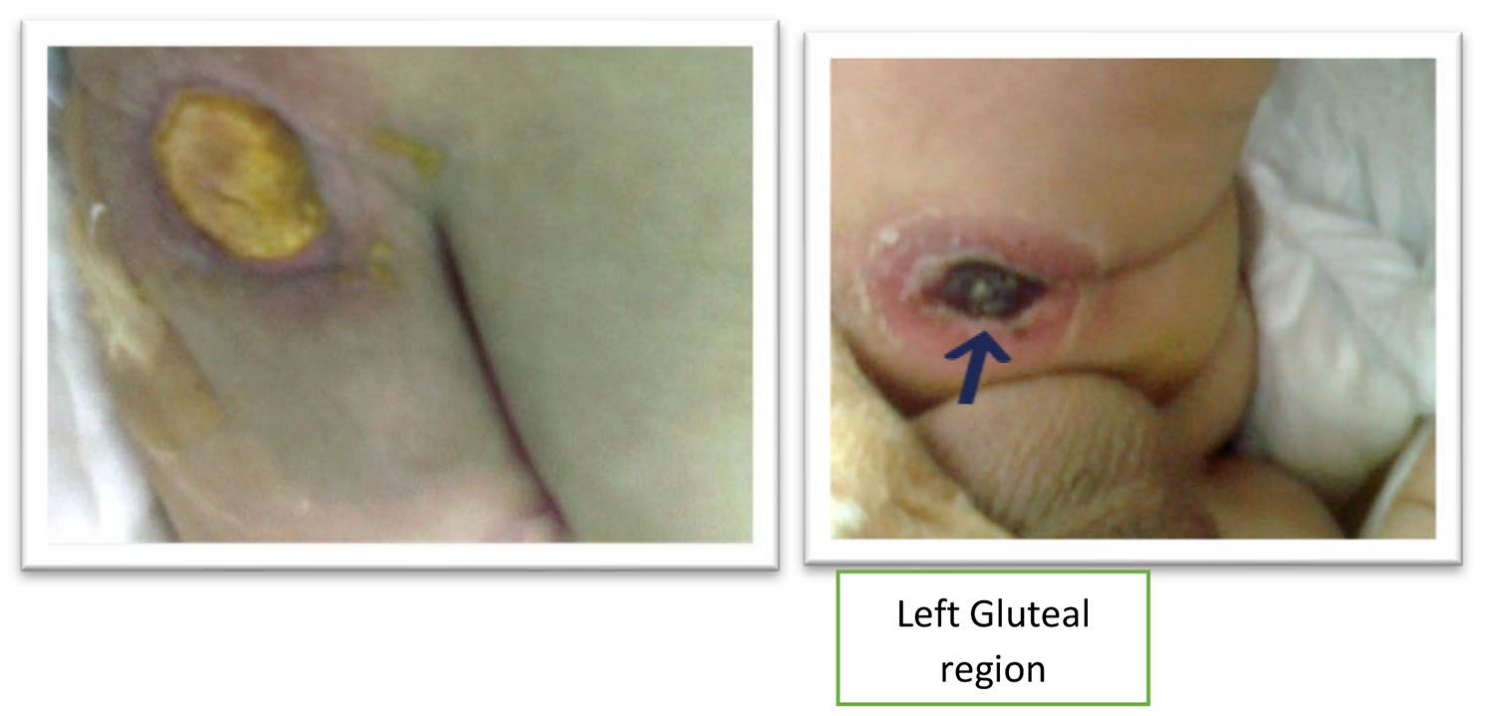
Non-healing ulcers on gluteal regions of patient

A four-month-old child presented with delayed wound healing post-circumcision and sepsis. During the hospital stay, he developed multiple necrotic lesions around the perineum, thighs, gluteal region, and neck (Fig. 1). On examination, he was anemic and febrile with a temperature of 39°C. There was no pus formation in and around the wounds and the penis had sloughed off. The wound swab culture grew Klebsiella species and Acinetobacter. During admission, the patient also developed an ear infection. Ear discharge and blood cultures grew pseudomonas. There was no hepatosplenomegaly or lymphadenopathy and no abnormality was found in the rest of the clinical examination, including the respiratory system, cardiovascular system, and nervous system.

Because of non-healing wounds and sepsis, a multidisciplinary action was taken involving infectious disease specialists and immunologists, and pediatric urologists.

Further history revealed that he was born of consanguineous marriage at full term by the caesarian section. He was the second child in the family and was breastfeeding. The umbilical cord of this child was separated on the 22nd day of birth and there was no omphalitis.

One elder sister died at the age of 2.5 years due to sepsis after receiving Bacillus Calmette Guerin (BCG) vaccination. According to the parents, she developed an infection of BCG scar which later on evolved into sepsis and gangrene of the hand, eventually causing the death of the child. Because of this family history and clinical presentation, this patient was not vaccinated.

**Fig. 2:**
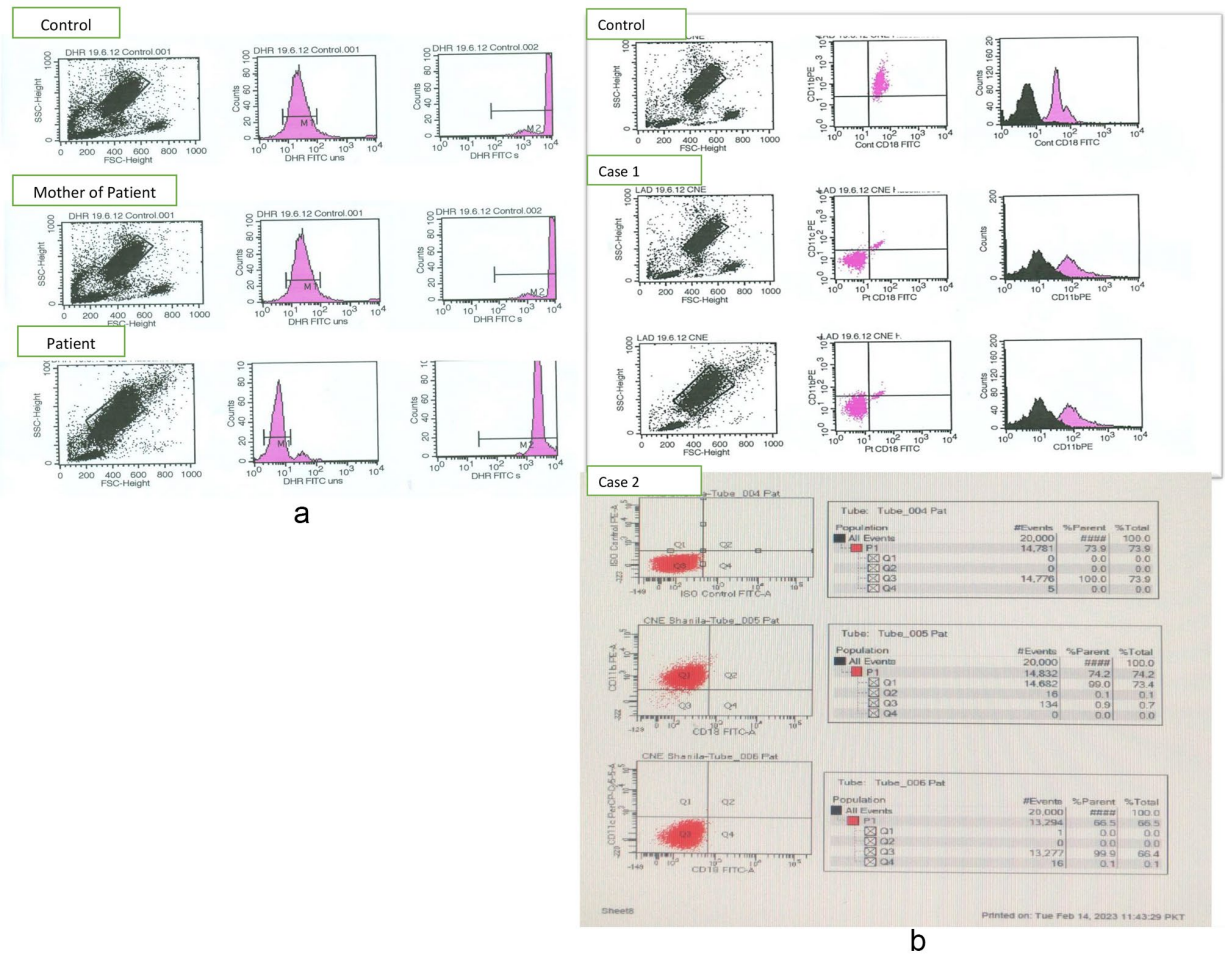
Flow cytometric analysis a: Dihydrorhodamine assay by flowcytometry showing normal oxidative burst by neutrophils There is a normal shift from baseline after stimulation in the patient compared to healthy control and the patient’s mother’s leucocytes. The Stimulation index of neutrophils after stimulation Vs. unstimulated cells were more than 100 in healthy control, mother, and patient. b: Flow cytometric analysis of leukocyte adhesion molecules (CD11b, CD11c, CD18). Expression of CD11b, CD11c and Cd18 was absent (<1%) on granulocytes in case 1, and in case 2, there is absence (<1%) of CD11c and CD18 the patient?s granulocytes, while expression of CD11b was 74%

Considering non-healing wounds and severe difficult-to-treat infections, and suggestive family history, a work-up for immunodeficiency was initiated. A preliminary suspicion of primary immunodeficiency included chronic granulomatous disease, IFN-γ-IL-12 axis defect, leukocyte adhesion deficiency (LAD), and Chediak Higashi syndrome. Other immunodeficiencies were also considered initially but with very low probability including cellular immunodeficiency and antibody deficiency. Initial investigations showed normal levels of immunoglobulin (IgG, IgM, and IgA) and slightly raised IgE (Table 1). His total leucocyte count was very high with relative neutrophilia. Accordingly, all lymphocyte subsets (T, B, and NK) were also increased (Table 1). The neutrophil function test was negative by both the nitroblue tetrazolium slide test (NBT) and dihydro rhodamine (DHR) assay by flow cytometry (Table 2 and Fig. 2a). Flow cytometric analysis for adhesion molecules showed a complete absence (< 1%) of CD11 and CD18 on granulocytes consistent with leucocyte adhesion deficiency type 1 (LAD1) (Fig. 2b). Therefore, the *ITGB2* gene was screened for a pathogenic gene variant. A homozygous likely pathogenic variant (c.817G > A; p. Val273Met) was identified confirming type 1 LAD.

**Table 1:** Laboratory test results a. Case 1

Age: four months				
Hematological Parameter			Reference ranges	
Hemoglobin level	8.6 g/dl		11.1–16.3 g/dl	
Total Leucocyte Count	119 x 10^3^ cells/µl		4–10 x 10^3^ cells/µl	
Neutrophils	82% (97.5 10^3^/ µl)		40–80% (2–7 x 10^3^ cells/µl)	
Lymphocytes	11% (13.1 10^3^/ µl)		20–40% (1–3 x 10^3^ cells/µl)	
Monocytes	7% (8.310^3^/ µl)		2–10% (0.2-1 10^3^ cells/µl)	
Platelets	94 x 10^3^/ µl		150-410x 10^3^ cells/µl	
Peripheral film:	Normochromic, leukocytosis with predominant neutrophilia		Normochromic, Normocytic	
Blood group:	B Positive			
Immunological Work-up			Reference range	
Serum Immunoglobulins (Ig)				
IgG	10.5		2.3 to 14.1 g/l	
IgA	1.19		0-0.83 g/l	
IgM	2.00		0-1.45 g/l	
IgE	272		< 100 IU/ml	
Neutrophil Function test:				
NBT^1^ slide test	No abnormality detected			
Dihydrorhodamine (DHR) Test	Stimulation Index (SI) = 400 (Control = 200)			
Lymphocyte subset analysis:	% Ratio	Cells/µl	% Ratio	Cells/µl
Total CD3^+^ T cells	72	8251	57 to 81	1000 to 4900
CD3^+^CD4^+^ (helper) T cells	46	5272	24 to 47	500 to 2700
CD3^+^CD8^+^ (cytotoxic) T cells	27	3094	17 to 37	300 to 2100
CD16^+^56^+^ (NK^2^) cells	14	1604	8 to 28	200 to 900
CD 19^+^ (B) cells	14	1604	10 to 27	200 to 2200
Flow cytometric analysis for CD11/CD18	expression of CD11b, CD11c, and CD18 was less than 1% on the patient’s neutrophils.			
Other tests:				
C-reactive Protein-H (CRP-H)	35 mg/dl		< 0.744 mg/dl	
Anti-HIV^3^-IgG	Negative		Negative	
Anti-HCV^4^-IgG	Negative		Negative	
Mutation analysis for beta 2 integrin gene	Homozygous variant in *ITGB2* (c.817G > A) gene, reported being pathogenic according to HGMD-public database.			

**Table 2:** Laboratory test results b. Case 2

Age: two years and eight months					
Hematological Parameter				Reference range	
Hemoglobin level	5.6 g/dl			11.1–16.3 g/dl	
Total Leucocyte Count	142 x 10^3^ cells/µl			4–10 x 10^3^ cells/µl	
Neutrophils	87% (124 x 10^3^ cells / µl)			40–80% (2–7 x 10^3^ cells/µl)	
Lymphocytes	10% (14.2x10^3^ cells / µl)			20–40% (1–3 x 10^3^ cells/µl)	
Monocytes	0			2–10% (0.2-1x 10^3^ cells/µl)	
Eosinophils	1% (1.4x10^3^ cells / µl)			1–6% (0.02–0.5 x 10^3^ cells/µl)	
Platelets	94 x 10^3^ cells / µl			150-410x 10^3^ cells/µl	
Peripheral film:	hypochromic, anisocytosis, leukocytosis with neutrophilia			Normochromic and normocytic	
Blood group:	O positive				
Immunological Work-up				Reference range	
Serum Immunoglobulins (Ig):					
IgG	9.5			2.3 to 14.1 g/l	
IgA	0.9			0-0.83 g/l	
IgM	1.5			0-1.45 g/l	
IgE	476			< 100 IU/ml	
Complements (C)					
C3	0.8			0.8–1.73 g/l	
C4	0.19			0.13–0.47 g/l	
Lymphocyte subset analysis:	% Ratio	Cells/µl		% Ratio	Cells/µl
Total CD3^+^ T cells	57	8580		57 to 81	1000 to 4900
CD3^+^CD4^+^ (helper) T cells	36.7	5580		24 to 47	500 to 2700
CD3^+^CD8^+^ (cytotoxic) T cells	17.5	2670		17 to 37	300 to 2100
CD16^+^56^+^ (NK^1^) cells	10.1	1540		8 to 28	200 to 900
CD 19^+^ (B) cells	31.4	4780		10 to 27	200 to 2200
Flow cytometric analysis for CD11/CD18	expression of CD11c, and CD18 were less than 1% on the patient’s neutrophils, while expression of CD11b was 74%				
Other tests:					
C-reactive Protein-H (CRP-H)	35 mg/dl		< 05 mg/dl		
Anti-HIV^2^-IgG	Negative		Negative		
Anti-HCV^3^-IgG	Negative		Negative		
Mutation analysis for beta 2 integrin gene	A homozygous splice-site variant (c.994–1G > C) was detected in the patient.				

Abbreviations: HCV = hepatitis C Virus, HIV = human immunodeficiency virus, NK = Natural killer, NBT = nitroblue tetrazolium

The patient was rigorously managed surgically and medically. Hemostatic suturing of bleeders was applied at the penile shaft and daily dressing was done. He was given broad-spectrum intravenous antibiotics and an injection of vitamin K. Blood and platelets were also transfused. He was subsequently discharged on oral antibiotics. At the time of discharge, his hemoglobin was 10.6 mg/dl, TLC was 59 × 10^3^ cells/ µl and platelets were 244 × 10^3^/µl. Parents were counseled to maintain strict hygiene, regular follow-up, and prenatal diagnosis in a subsequent pregnancy. They have also advised bone marrow transplantation of their child.

#### Two years and eight months old

Two years and eight-month-old female child presented with right eye swelling. It started as a pustule on the right eyelid two months back. There is an associated high-grade fever. On examination, the patient was febrile and irritable. The right eye was red and swollen and almost closed with a scab formation. The chest was clear and the abdomen was soft with no visceromegaly but there was a reducible umbilical hernia. CT scan and MRI brain findings were consistent with a large heterogenous abscess in the inferior margin of the right globe extending into the right intraconal component which can be due to chronic granulomatous infections such as mycobacterium tuberculosis (MTB) or fungal infections The lesion did not involve maxillary bones and was confined to the soft tissues of the orbit. Paranasal sinuses were clear. An excision biopsy of the lesion confirmed abscess formation but was negative for fungal infections. The smear was negative for acid-fast bacilli and MTB was not detected in the pus by GeneXpert testing. Cultures from the wound showed Pseudomonas aeruginosa. Blood cultures were negative. The child was put on ciprofloxacin and voriconazole.

Further history revealed delayed shedding of the umbilical cord at one month of age but there was no omphalitis. She was born of consanguineous marriage and was vaccinated only for BCG after which she developed a high-grade fever. Family history was significant for a death of a sibling (male) at 8 months of age 5 years back due to sepsis. He had omphalitis and a severe reaction to BCG vaccination. One sister (7 years) and one brother (2 years) are alive and healthy.

**Fig. 3:**
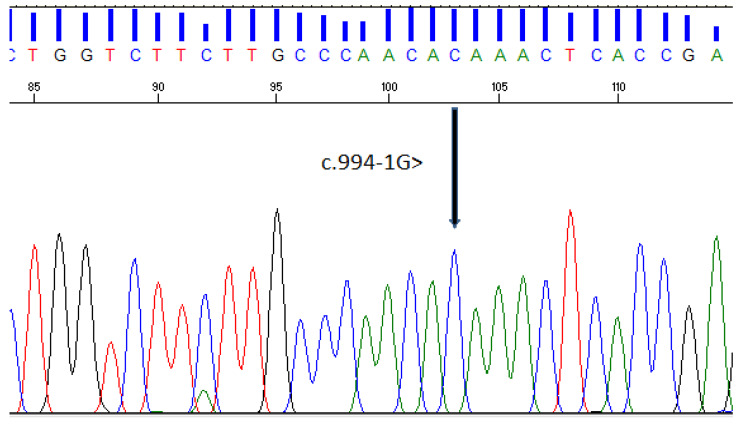
ITGB2 gene screening for Leukocyte adhesion deficiency type 1 showing c.994‒1G > C variant

Complete blood counts showed a very high TLC (142 × 10^3^/µl) with predominant neutrophilia. Considering the significant history of infections, reaction to BCG vaccination, family history of the death of a sibling, and a very high TLC count, an immunodeficiency workup was initiated. Serum Immunoglobulins and complement levels (C3 and C4) were normal. Lymphocyte subset analysis showed high counts of all lymphocytes including T cells, B cells, and NK cells. (Table 1b). Flow cytometric analysis for CD11/CD18 showed a complete absence of CD11c and CD18 (< 1%) on the patient’s granulocytes (Fig. 2b). Genetic analysis showed a novel homozygous splice-site pathogenic variant (c.994‒1G > C), consistent with the diagnosis of leukocyte adhesion deficiency type 1 (LAD 1) (Table 2 and Fig. 3).

The patient was treated vigorously for infections with vancomycin, voriconazole, and cefixime. Her wound became slightly better, her fever subsided and TLC counts dropped to 50 × 10^3^/µl.

## Discussion and conclusions

PIDs are very rare disorders with heterogeneous manifestations. The main clinical features of these disorders are recurrent severe infections, and the diagnosis is often missed or delayed. Moreover, genetic testing is often required to make a definite diagnosis [3, 4].

PIDs are classified according to the defects in cells or molecules and the resulting phenotypes [1]. LAD is characterized by severe and recurrent infections due to defective chemotaxis of leucocytes. The migration of leucocytes along blood vessel walls and their emigration to the site of injury involves various sets of adhesion molecules. These molecules are expressed on both resting and stimulated endothelial cells and leucocytes. Depending upon the defect in the expression of different adhesion molecules, LAD is categorized into LAD I to III. In LAD I there is a defect in ß2 integrins on leucocytes, In LAD II, there is the absence of fucosylated ligands for selectins, and in LAD III, ß2 integrins are present but functionally defective [5, 8].

The defect in LAD I is due to biallelic loss of the *ITGB2* gene, encoding the β2 subunit of integrin. This leads to decreased expression of CD11a (αLβ2, LFA-1), CD11b (αMβ2, Mac-1), CD11c (αXβ2), and CD11d (αDβ2) integrins on leucocytes that hampers their migration to the site of infection or inflammation in the tissues from blood [3, 8, 12]. As a result, there are recurrent infections, especially bacterial and fungal infections of varying severity, non-healing wounds without pus formation, and early death usually in infancy [3, 8]. There is often a history of delayed separation of the umbilical cord and omphalitis [3, 8, 11–13]. More than 150 gene variants have been identified in patients with LAD1 (OMIM # 600,065) including gross deletion, insertion, nonsense, and missense variants, etc. that were usually found pathogenic [6, 11, 21–23]. The product of this gene belongs to the integrin beta chain family of proteins, known to participate in cell adhesion as well as cell-surface mediated signaling. The variant c.817G > A; p.G273R identified in the first case has been reported previously in a patient with a moderately severe phenotype of LAD-1 [24]. The variant is located in the conserved B2 subunit domain leads to the non-expression of the integrin molecule and affects heterodimer formation. Another likely pathogenic splice-site variant (c.994‒1G > C) in the *ITGB2* gene is found in patient 2 (Table 2; Fig. 3). NetGene2 v. 2.4 (http://www.cbs.dtu.dk/services/NetGene2/) bioinformatics tools were employed to assess the pathogenicity of this variant that termed this variant as pathogenic. This variant abolishes the splice junction for exon 8 in the *ITGB2* gene and is predicted to cause loss of function. The variant is novel and not reported in the literature or HGMD (Human Gene Mutation Database; http://www.hgmd.org/) but reported in the ClinVar database. This variant is observed in 1/30,616 (0.0032%) alleles from individuals of South Asian background in gnomAD Exomes and is novel (not in any individuals) in 1000 Genomes databases. According to the ACMG guidelines, the c.994‒1G > C variant is classified as a pathogenic disease-causing. The criteria applied were PVS1 (very strong) applied for null variant (intronic within ± 2 of splice site) in *ITGB2* gene, PP5 (moderate) ClinVar classifies this variant as likely pathogenic and PM2 (supporting) either not identified in gnomAD genomes or homozygous allele count less than 2 in gnomAD genomes.

It should be remembered that PIDs have heterogeneous manifestations and therefore requires expertise to recognize these disorders. LADs have a very low prevalence with less than 400 cases reported to date adding to diagnostic dilemmas [14, 15].

In the first case, there was a diagnostic dilemma with impaired wound healing post-circumcision which could have easily been blamed on the surgeon’s unskilled approach. However, a very high TLC count, multiple subsequent infections, and difficult-to-treat wounds led to suspicion of primary immunodeficiency.

Various Eye involvement is known with primary immunodeficiency disorders including antibody deficiencies, severe combined immunodeficiency, neutrophil function defects, complement proteins abnormality, CHARGE syndrome, etc. [16]. In LAD, this is not a typical manifestation and there are only case reports showing necrotizing infection of the eyeball as the presenting feature [17, 18]. However, staphylococcus aureus, pseudomonas, and fungi are common pathogens found in LAD patients [19, 20]. Because of the rarity of this manifestation in LAD, the diagnosis in the second case may have been missed. But a careful history taking into account the consanguinity and a positive family history with a very high leucocyte count triggered the suspicion and further workup for this disorder.

In a Chinese cohort of seven patients, novel mutations were identified in four [25]. Tipu et al. [15] have reported a genetic analysis of 12 Pakistani patients with five pathogenic variants in eight cases and one of the identified variants was novel.

The significance of these case reports lies in the clinical presentation of these children and clues leading to definite diagnoses which otherwise were diagnostic dilemmas. In both cases, there was no omphalitis but there were severe recurrent infections and reactions to BCG vaccination either in the patient or in the siblings who succumbed to intractable infections.

We conclude that in both, cases, the most important lesson learned was a multidisciplinary approach and a low threshold to suspect primary immunodeficiency that leads to a better understanding of the problem. Not only that, the family could be counseled to seek proper treatment and precautionary measures to avoid infections and complications and opt for bone marrow transplantation.

## Data Availability

All data generated during the study are presented in this article.

## List of Abbreviations

BCG: Bacillus Calmette Guérin
CFTR: Cystic fibrosis transmembrane conductance regulator
CT: Computed tomography
DHR: Dihydrorhodamine
FERMT3: Fermitin family homologue 3
IFN-γ: interferon gamma
IL-12: Interleukin-12
IHHN: Indus Hospital and Health network
ITGB2: integrin subunit beta2
LAD: Leukocyte adhesion defect
LFA-1: Lymphocyte Function Associated Antigen 1
NBT: Nitroblue tetrazolium test
PID: Primary immunodeficiency
SIUT: Sindh Institute of Urology and Transplantation
TLC: total leukocyte count

## References

[1] Tangye SG, Al-Herz W, Bousfiha A, Chatila T, Cunningham-Rundles C, Etzioni A, Franco JL, Holland SM, Klein C, Morio T, Ochs HD, Oksenhendler E, Picard C, Puck J, Torgerson TR, Casanova JL, Sullivan KE. Human Inborn Errors of Immunity: 2019 Update on the Classification from the International Union of Immunological Societies Expert Committee. J Clin Immunol. 2020 Jan;40(1):24–64. DOI: 10.1007/s10875-019-00737-x. Epub 2020 Jan 17. Erratum in: J Clin Immunol. 2020 Feb 22; PMID: 31953710; PMCID: PMC7082301. 10.1007/s10875-019-00737-x PMC7082301 31953710

[2] Pilania RK, Chaudhary H, Jindal AK, Rawat A, Singh S. Current status and prospects of primary immunodeficiency diseases in Asia. Genes Dis. 2019 Sep 12;7(1):3–11. DOI: 10.1016/j.gendis.2019.09.004. PMID: 32181271; PMCID: PMC7063407 10.1016/j.gendis.2019.09.004 PMC7063407 32181271

[3] Das J, Sharma A, Jindal A, Aggarwal V, Rawat A. Leukocyte adhesion defect: Where do we stand circa 2019? Genes Dis.2019 Aug7;7(1):107–114. DOI: 10.1016/j.gendis.2019.07.012. PMID: 32181281; PMCID: PMC7063431. 10.1016/j.gendis.2019.07.012 PMC7063431 32181281

[4] Nigar S, Khan EA, Ahmad TA. Leukocyte adhesion defect: an uncommon immunodeficiency. J Pak Med Assoc. 2018 Jan;68(1):119–22. PMID: 29371732. 29371732

[5] Etzioni A. Defects in the leukocyte adhesion cascade. Clin Rev Allergy Immunol. 2010 Feb;38(1):54–60. DOI: 10.1007/s12016-009-8132-3. PMID: 19437145. 10.1007/s12016-009-8132-3 19437145

[6] Kambli PM, Bargir UA, Yadav RM, Gupta MR, Dalvi AD, Hule G, Kelkar M, Sawant-Desai S, Setia P, Jodhawat N et al. M. Clinical and Genetic Spectrum of a Large Cohort of Patients with leukocyte adhesion deficiency Type 1 and 3: A multicentric study from India.Front Immunol. 2020 Dec16; 11:612703. DOI: 10.3389/fimmu.2020.612703. PMID: 33391282; PMCID: PMC7772426. 10.3389/fimmu.2020.612703 PMC7772426 33391282

[7] Etzioni A. Genetic etiologies of leukocyte adhesion defects. Curr Opin Immunol. 2009 Oct;21(5):481-6. DOI: 10.1016/j.coi.2009.07.005. Epub 2009 Aug 3. PMID: 19647987. 10.1016/j.coi.2009.07.005 19647987

[8] van de Vijver E, Maddalena A, Sanal Ö, Holland SM, Uzel G, Madkaikar M, de Boer M, van Leeuwen K, Köker MY, Parvaneh N, Fischer A, Law SK, Klein N, Tezcan FI, Unal E, Patiroglu T, Belohradsky BH, Schwartz K, Somech R, Kuijpers TW, Roos D. Hematologically important mutations: leukocyte adhesion deficiency (first update).Blood Cells Mol Dis. 2012 Jan15;48(1):53–61. DOI: 10.1016/j.bcmd.2011.10.004. Epub 2011 Nov 30. PMID: 22134107; PMCID: PMC4539347. 10.1016/j.bcmd.2011.10.004 PMC4539347 22134107

[9] Fan Z, Ley K, Leukocyte Adhesion Deficiency IV. Monocyte Integrin Activation Deficiency in cystic fibrosis. Am J Respir Crit Care Med. 2016 May;15(10):1075–7. 10.1164/rccm.201512-2454ED. PMID: 27174474; PMCID: PMC4872669. 10.1164/rccm.201512-2454ED PMC4872669 27174474

[10] Movahedi M, Entezari N, Pourpak Z, Mamishi S, Chavoshzadeh Z, Gharagozlou M, Mir-Saeeid-Ghazi B, Fazlollahi MR, Zandieh F, Bemanian MH, Farhoudi A, Aghamohammadi A. Clinical and laboratory findings in Iranian patients with leukocyte adhesion deficiency (study of 15 cases). J Clin Immunol. 2007 May;27(3):302-7. DOI: 10.1007/s10875-006-9069-4. Epub 2007 Feb 10. Erratum in: J Clin Immunol. 2008 Jan;28(1):92. Aghamohammadi, Asghar [added]. PMID: 17294145. 10.1007/s10875-006-9069-4 17294145

[11] Yaz I, Ozbek B, Bildik HN, Tan C, Oskay Halacli S, Soyak Aytekin E, Esenboga S, Cekic S, Kilic SS, Keskin O, van Leeuwen K, Roos D, Cagdas D, Tezcan I. Clinical and laboratory findings in patients with leukocyte adhesion deficiency type I: a multicenter study in Turkey. Clin Exp Immunol. 2021 Oct;206(1):47–55. 10.1111/cei.13645. Epub 2021 Aug 5. PMID: 34310689; PMCID: PMC8446394. 10.1111/cei.13645 PMC8446394 34310689

[12] De Rose DU, Giliani S, Notarangelo LD, Lougaris V, Lanfranchi A, Moratto D, et al. Long term outcome of eight patients with type 1 leukocyte Adhesion Deficiency (LAD-1): not only infections, but high risk of autoimmune complications. Clin Immunol. 2018 Jun;191:75–80. Epub 2018 Mar 13. PMID: 29548898. 10.1016/j.clim.2018.03.005 29548898

[13] Wolach B, Gavrieli R, Wolach O, Stauber T, Abuzaitoun O, Kuperman A, Amir Y, Stepensky P, Somech R, Etzioni A. Leucocyte adhesion deficiency-A multicentre national experience. Eur J Clin Invest. 2019 Feb;49(2): e13047. DOI: 10.1111/eci.13047. Epub 2019 Jan 4. PMID: 30412664. 10.1111/eci.13047 30412664

[14] Almarza Novoa E, Kasbekar S, Thrasher AJ, Kohn DB, Sevilla J, Nguyen T, Schwartz JD, Bueren JA. Leukocyte adhesion deficiency-I: A comprehensive review of all published cases. J Allergy Clin Immunol Pract. 2018 Jul-Aug;6(4):1418–1420.e10. DOI: 10.1016/j.jaip.2017.12.008. Epub 2018 Jan 20. PMID: 29371071. 10.1016/j.jaip.2017.12.008 29371071

[15] Nawaz Tipu H, Raza R, Jaffar S, Khan A, Anwar MZ, Ahmad W, Raza SI. β2 Integrin Gene (ITGB2) mutation spectra in Pakistani families with leukocyte adhesion deficiency type 1 (LAD1). Immunobiology. 2020 May;225(3):151938. doi: 10.1016/j.imbio.2020.151938. Epub 2020 Apr 2. PMID: 32279896. 10.1016/j.imbio.2020.151938 32279896

[16] Hosseinverdi S, Hashemi H, Aghamohammadi A, Ochs HD, Rezaei N. Ocular involvement in primary immunodeficiency diseases. J Clin Immunol. 2014 Jan;34(1):23–38. 10.1007/s10875-013-9974-2. Epub 2013 Nov 30. PMID: 24292697. 10.1007/s10875-013-9974-2 24292697

[17] Gupta V, Pandita A, Panghal A, Pillai A. Leucocyte adhesion defect presenting as fulminant sepsis in a new born. BMJ Case Rep. 2019 Aug 30;12(8):e227065. doi: 10.1136/bcr-2018-227065. PMID: 31471353; PMCID: PMC6720668. 10.1136/bcr-2018-227065 PMC6720668 31471353

[18] Ganesh A, Al-Zuhaibi SS, Bialasiewicz AA, Al-Abri R, Ahmed S, Al-Tamemi S, El-Nour IB. Necrotizing Pseudomonas infection of the ocular adnexa in an infant with leukocyte adhesion defect. J Pediatr Ophthalmol Strabismus. 2007 Jul-Aug;44(4):199–200. doi: 10.3928/01913913-20070701-09. PMID: 17694822. 10.3928/01913913-20070701-09 17694822

[19] Rafiei Tabatabaei S, Karimi A, Amanati A, Kazemi Aghdam M, Shamsian BS (2013). Diagnostic dilemma in a patient with chronic fistulating nonhealing Ulcer. Arch Pediatr Infect Dis.

[20] Thakur N, Sodani R, Chandra J, Singh V. Leukocyte adhesion defect type 1 presenting with recurrent pyoderma gangrenosum. Indian J Dermatol. 2013 Mar;58(2):158. 10.4103/0019-5154.108076. PMID: 23716823; PMCID: PMC3657233. 10.4103/0019-5154.108076 PMC3657233 23716823

[21] Hixson P, Smith CW, Shurin SB, Tosi MF. Unique CD18 mutations involving a deletion in the extracellular stalk region and a major truncation of the cytoplasmic domain in a patient with leukocyte adhesion deficiency type 1. Blood. 2004 Feb 1;103(3):1105-13. doi: 10.1182/blood-2003-08-2780. Epub 2003 Sep 25. PMID: 14512306. 10.1182/blood-2003-08-2780 14512306

[22] Guan S, Tan SM, Li Y, Torres J, Uzel G, Xiang L, Law SK. Characterization of single amino acid substitutions in the β2 integrin subunit of patients with leukocyte adhesion deficiency (LAD)-1. Blood Cells Mol Dis. 2015 Feb;54(2):177–82. doi: 10.1016/j.bcmd.2014.11.005. Epub 2014 Nov 28. PMID: 25514840. 10.1016/j.bcmd.2014.11.005 25514840

[23] Hong S, Xie LJ, Yang QN, Zhu TW (2018). Detection of leukocyte adhesion deficiency type 1 in an infant by highthroughput targeted exome sequencing. J Transl Genet Genom.

[24] Hogg N, Stewart MP, Scarth SL, Newton R, Shaw JM, Law SK, Klein N (1999). A novel leukocyte adhesion deficiency caused by expressed but nonfunctional beta2 integrins Mac-1 and LFA-1. J Clin Invest.

[25] Sun B, Chen Q, Dong X, Liu D, Hou J, Wang W, Ying W, Hui X, Zhou Q, Yao H, Sun J, Wang X. Report of a Chinese Cohort with Leukocyte Adhesion Deficiency-I and Four Novel Mutations.J Clin Immunol. 2019Apr;39(3):309–315. doi: 10.1007/s10875-019-00617-4. Epub 2019 Mar 27. PMID: 30919141. 10.1007/s10875-019-00617-4 30919141

